# MicroRNA-196a is regulated by ER and is a prognostic biomarker in ER+ breast cancer

**DOI:** 10.1038/s41416-019-0395-8

**Published:** 2019-02-20

**Authors:** Michael J. G. Milevskiy, Udai Gujral, Carolina Del Lama Marques, Andrew Stone, Korinne Northwood, Lez J. Burke, Julia M. W. Gee, Kenneth Nephew, Susan Clark, Melissa A. Brown

**Affiliations:** 1 School of Chemistry and Molecular Biosciences, University of Queensland, St Lucia, QLD Australia; 2 Division of Genomics and Epigenetics, Epigenetics Research Laboratory, Garvan Institute of Medical Research, Sydney, NSW Australia; 3 UQ Centre for Clinical Research, The University of Queensland, Herston, QLD Australia; 4 School of Pharmacy and Pharmaceutical Sciences, Cardiff University, Cardiff, UK; 5 School of Medicine, Indiana University, Bloomington, IN USA; 6 Present Address: ACRF Stem Cells and Cancer, The Walter and Eliza Hall Institute of Medical Research, Parkville, VIC Australia

**Keywords:** Breast cancer, Transcriptional regulatory elements, miRNAs

## Abstract

**Background:**

MicroRNAs are potent post-transcriptional regulators involved in all hallmarks of cancer. *Mir-196a* is transcribed from two loci and has been implicated in a wide range of developmental and pathogenic processes, with targets including Hox, Fox, Cdk inhibitors and annexins. Genetic variants and altered expression of *MIR196A* are associated with risk and progression of multiple cancers including breast cancer, however little is known about the regulation of the genes encoding this miRNA, nor the impact of variants therein.

**Methods:**

Genomic data and chromatin interaction analysis were used to discover functional promoter and enhancer elements for *MIR196A*. Expression data were used to associate *MIR196A* with mechanisms of resistance, breast cancer subtypes and prognosis.

**Results:**

Here we demonstrate that *MIR196A* displays complex and dynamic expression patterns, in part controlled by long-range transcriptional regulation between promoter and enhancer elements bound by ERα. Expression of this miRNA is significantly increased in drug-resistant models of hormone-receptor positive disease. The expression of *MIR196A* also proves to be a robust prognostic factor for patients with advanced and post-menopausal ER+ disease.

**Conclusion:**

This work sheds light on the normal and abnormal regulation of *MIR196A* and provides a novel stratification method for therapeutically resistant breast cancer.

## Background

MicroRNAs are short non-coding RNAs that post-transcriptionally regulate gene expression.^1^ MicroRNAs have been implicated in many diseases, including rare inherited syndromes, arising from germline mutations in MiRNA genes, and several cancers types.^2^ Research into the biology and pathology of these molecules has led to the identification of clinically useful genetic and epigenetic biomarkers and more recently novel therapeutic agents.^3^ These therapeutic agents are based on antagomiR technology, synthetic RNA molecules that bind miRNA targets, and have shown promise in the control of disease symptoms and progression.

*MicroRNA-196A* (mature RNA *MIR196A*, non-human miR196a) is transcribed from two genomic loci, *HOXC* (Chr12 in humans, gene *MIR196A2*) and *HOXB* (Chr17 in humans, gene *MIR196A1*), both situated upstream of *HOX9*, respectively.^4^ The precursor transcript expressed from *MIR196A2* (*pre-MIR196A2*) produces two mature miRNA molecules, miR-196a-5p (herein referred to as *MIR196A, miR196a* non-human) and miR-196a-3p, whilst the *HOXB* precursor gene *MIR196A1* (*pre-MIR196A1*) also encodes miR-196a-5p but a different 3′ miRNA, miR-196a-1-3p. Early studies into the function of *miR196a* in mice and chicken, demonstrated a requirement for *miR196a* expression to suppress Hoxb8 RNA, essentially controlling its spatiotemporal pattern along the anterior-posterior axis.^5–8^

*MIR196A* been implicated in a range of cancers, primarily as an oncogene. For example, *MIR196A* is overexpressed in breast tumours relative to normal breast tissue,^9^ and additionally a single nucleotide polymorphism (SNP, rs11614913, C>T) within the *MIR196A2* gene is associated with a decreased risk of breast cancer.^10^ The decrease in risk from rs11614913 was found to be associated with a decrease in processing of the precursor transcript to mature miRNA, resulting in less *MIR196A* expression and highly suggestive of an oncogenic role in breast cancer. *MIR196A* has also been shown to target the 3′ UTR of Annexin-1 (*ANXA1*), an important mediator of apoptosis,^11^ in response to the pro-angiogenic vascular endothelium growth factor (VEGF), leading to alterations in angiogenesis, a hallmark of tumourigenesis.^12^ A separate study demonstrated that *MIR196A* could increase growth, migration and invasion of a non-small-cell lung cancer cell line through direct targeting of *HOXA5*.^13^ Two studies have recently shown that *MIR196A* can directly influence the cell cycle by targeting p27/Kip1, an inhibitor of cell cycle progression, to dramatically increase growth and pro-oncogenic features of cancer cell lines.^14,15^ Despite the clear importance on *MIR196A* in cancer, its transcriptional regulation remains poorly understood.

Transcriptional regulation is a complex multi-faceted biological process that is significantly altered in cancer. MicroRNA genes are regulated transcriptionally in a similar manner to protein coding and long non-coding RNA genes. Promoters mostly lie upstream (within 10 kb of the mature miRNA), contain a CpG island and in an active state when the miRNAs are transcribed by RNA Pol II are enriched for H3K4me3 and lack H3K27me3 similar to protein coding genes.^16,17^ Taken together, these data indicate that potential promoters for miRNAs can be identified in a similar manner to methods for protein coding genes. Several instances of miRNA regulation by enhancers have been described, but this area is very much in its infancy.^18,19^

In this study, we aimed to characterise the expression landscape of *MIR196A* including factors regulating its expression and explore potential roles of regulatory elements and factors in breast cancer prognostication.

## Material and methods

### Cell culture

MCF7 cells, for the development of endocrine resistance sub-lines were obtained from AstraZeneca. MCF7, Tamoxifen-resistant (TAMR), Fulvestrant-resistant (FASR), and oestrogen-deprived (MCF7x) cells were cultured as described.^20–22^ All cell lines were cultured for less than 6 months after authentication by short-tandem repeat (STR) profiling (Cell Bank, Australia). MCF7 cells were cultured in RPMI (ThermoFisher, 11875-093) supplemented with 5% foetal calf serum (FCS, ThermoFisher, 1600–044). TAMR, FASR and MCF7x lines were cultured in phenol-red free RPMI (ThermoFisher, 11835–030) supplemented with 5% charcoal stripped FCS (Sigma-Aldrich, F6765), additionally TAMR cells were maintained in 4-Hydroxytamoxifen (Sigma H7904, 10^−7^M) and FASR cells in Fulvestrant (Herceptin^®^, Genentech, 10^−7^M).

### Cloning and reporter assays

All PCR products for luciferase reporter assays were ligated into Invitrogen’s pCR-Blunt (K270020) plasmid using T4 DNA Ligase (New England BioLabs, M0202S), at 4^0 ^C overnight. *MIR196A* enhancers and promoters were digested from pCR-Blunt and cloned into the luciferase reporter plasmid pGL3-Basic (Promega, E1751). Enhancers were cloned into the *Bam*HI/*Sal*I site whilst promoters were cloned into the multiple cloning site immediately upstream of the luciferase gene. See Supplementary Table 1 for primers.

MCF7 cells were transfected in antibiotic free media with 500 ng of modified pGL3 reporter constructs, 20 ng of pRL-TK (Renilla transfection control) and with 0.5 μL of Lipofectamine 3000 (Life Technologies, L3000-008). 48 h post transfection luciferase readings were measured using a DTX-880 luminometer and Dual-Glo Stop and Glo luciferase reporter kit (Promega, E2920), following the manufacturer’s recommended protocol.

### RNA extraction and gene expression

Cell lysates were prepared using Life Technologies TRIzol® reagent and RNA was chloroform extracted and isopropanol precipitated. RNA was DNaseI treated with the DNA free kit from Ambion (Life Technologies, AM1906). RNA for miRNA analysis was reverse transcribed using the miScript RT II kit from Qiagen (218161), following instructions as per the manufacturer. Assays for all miRNAs were performed with Qiagen’s miScript SYBR Green PCR Kit (218073). Primers specific to each mature or precursor miRNA were assayed coupled with a universal primer, see Supplementary Table 2 for assay IDs. Expression data for miRNAs was normalised to the snoRNA RNU6b. All qRT-PCRs were performed using the protocols advised by the manufacturers on a Corbet Rotorgene-6000.

Processed read counts for RNA-Seq on MCF7 cells following oestradiol treatment was sourced from K. Nephew (see author list).^23^ RNA-Seq from Adriamycin (ADM) and paclitaxel (PTX) resistant MCF7 derived cells was sourced from GSE68815,^24^ as processed and normalised read counts. Expression of *HOX* genes in human breast cells was sourced from Gascard et al.^25^ as normalised read counts.

### Genomic data analysis

Accession codes for publicly available data were as follows, MCF7 ChIP-Seq (GSE14664,^26^), GRO-Seq (GSE27463,^27^), ChIA-PET (GSE39495,^28,29^), Breast tumour ERα ChIP-Seq (GSE32222,^30^). MCF7 histone ChIP-Seq and breast cell 450 K array data was sourced from ENCODE^31^ via http://genome.ucsc.edu/ENCODE/downloads.html. ChIP-Seq reads were adapter trimmed and data was mapped to the human genome (hg19) using Bowtie^32^ and peaks called by MACS^33^ and viewed in the Interactive Genome Viewer (IGV)^34^ available through the Broad Institute servers. DNA methylation 450 K array data for MCF7 and endocrine resistant sublines was previously published, see Stone et al.^35^ Normalised DNA methylation of breast tumours was sourced from The Cancer Genome Atlas (TCGA).^36^ Methylation β-values were correlated to the gene expression of *MIR196A* from the TCGA cohort,^36^ Pearson correlation coefficients are reported. For transcription factor (TF) binding to the *MIR196A2* promoters, the genomic regions upstream of the *MIR196A2* gene were visualised through the UCSC genome browser.^37^ ENCODE^38^ TF ChIP-Seq and JASPAR^39^ TF motifs were mapped across the putative promoter elements and snapshots from the browser were taken.

### Breast tumour expression analysis

METABRIC expression and clinical information were sourced from EGAS00000083 through a Material Transfer Agreement with the consortium.^40,41^ Expression values were pre-processed by METABRIC and available as log2 array intensities. Clustering of Illumina Array and miR-Seq data was performed using the Multiple Experiment Viewer (MeV,^42^). Data was mean-centred and hierarchically clustered via Manhattan average-linkage based clustering of both rows and columns. Genes were correlated within clusters using the CORREL function of Microsoft Excel. The protein network was generated through the cBioPortal link (www.cbioportal.org,^43^) using the TCGA data.^36^ cBioPortal utilises protein data and visualisation tools through Cytoscape.^44^

### Survival analysis

Tumour cohorts were based on immunohistochemistry of METBARIC^40^ patients as either ER+ or ER− PGR− HER2− as triple negative breast cancer (TNBC). Univariate and multivariate Cox proportional hazard regression analyses were performed using MedCalc for Windows, version 12.7 (MedCalc Software, Ostend, Belgium). Kaplan-Meier survival analysis and generation of survival curves was done in GraphPad Prism. Optimal cutoffs for low and high expression groups were determined using receiver operator characteristic (ROC) curves based on the expression of genes (*MIR196A, HER2* and *PGR*) versus patient overall survival. The gene expression value that represents the maximum deviation from the ‘random guess’ line was used as a cut-off to discriminate low versus high expression. Lymph node status was designated as positive (+, ≥1 node presenting with disease at time of surgery) or negative (−). Tumour grade and stage clinical information were sourced from METABRIC.^40^ Tumour size was categorised as T1 = ≤ 20 mm, T2 = > 20 mm, and <50 mm and T3 = ≥ 50 mm.

### 3C and ChIA-PET

Chromosome conformation capture (3C) was adapted from Vakoc 2005,^45^ Hagege 2007^46^ and Tan-Wong 2008.^47^ Briefly, cells were grown to 60–80% confluencey and fixed with 1% paraformaldehyde. Libraries were generated for each cell line using *Hind*III with control libraries undigested and unligated, representing native gDNA without chromosome conformation. GAPDH primers (amplified fragment contains no cut sites for these enzymes) were used to determine the digestion and ligation efficiency of each library by comparing 3C-qPCR values to primers that amplify a fragment containing a *Hind*III cut site. For each 3C-qPCR, primers were designed between 100–250 bp up or downstream of each *Hind*III cut site with the primer across the putative enhancer used as bait in each 3C-qPCR. The bait primer was combined with each of the primers across the enhancer region for the 3C-qPCR and Ct levels from each 3C-qPCR were normalised to the lowest Ct value (most abundant interaction) so that this interaction = 1 relative interaction. Enhancer-promoter interactions are demonstrated as a peak across a region of multiple primers.

## Results

### MIR196A expression correlates with HOXC genes in breast cancer

Several *HOXC* protein coding and non-coding genes have known associations with breast cancer progression. We assessed expression patterns of *HOXC* genes and *MIR196A* (mature miRNA) in the METABRIC cohort of breast tumours (Supp Fig. 1). These data indicate that *MIR196A* expression highly correlates to *HOXC* genes, particularly *HOXC10*, which lies directly upstream of this miRNA.

Next, we investigated whether these associations are also observed in normal cells of the human breast. Here associations between mature *MIR196A* expression and *HOXC* genes are more limited, with correlations most strongly with *HOXC11* and *HOXC10*, the genes upstream of the HOXC *MIR196A2* gene (Supp Fig. 2A). Consistent with its role in degrading HOX transcripts, *HOXC8*, *HOXB8* and *HOXA7* (all validated targets) negatively correlate with *MIR196A* expression. *MIR196A* appears to be most highly expressed within the basal stem-cell (BSC) derived cells, whilst expression is lower in the more differentiated cell types (Supp Fig. 2B).

### MIR196A expression is regulated by oestrogen

**Fig. 1:**
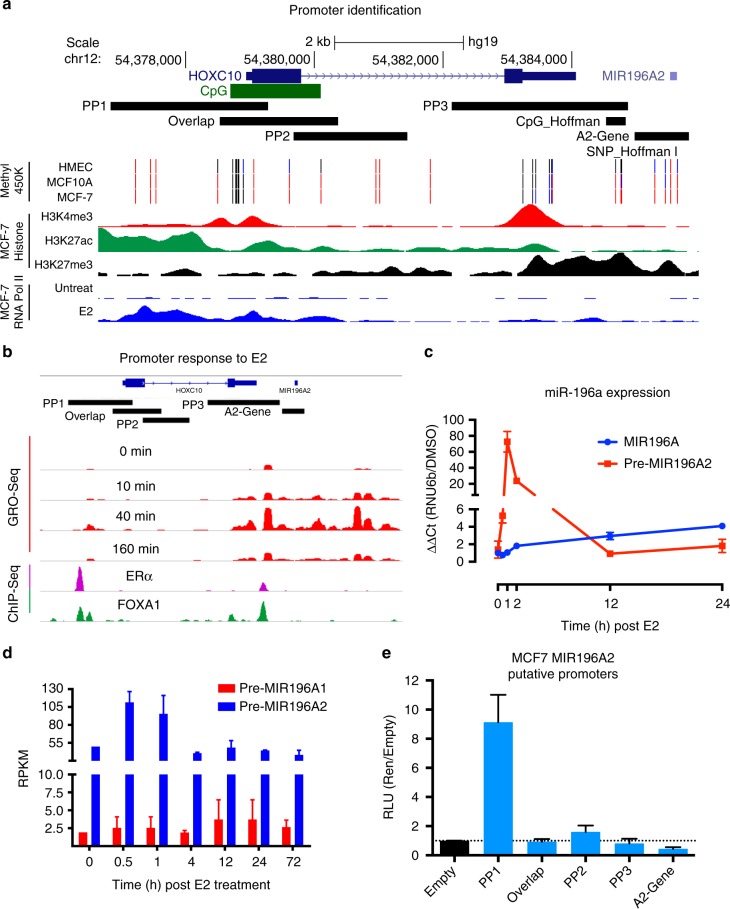
E2 influences *MIR196A2* expression in breast cancer. **a** Identification of putative promoter regions for the *MIR196A2* gene using histone marks and ChIP-Seq indicated in figure. Refseq genes are indicated in blue at the top with coordinates based on hg19 chromosome 12. Putative promoter regions (PP1,2,3) and the previously implicated SNP (rs116149130) and CpG from Hoffman et al.^10^ are indicated by black rectangles. MCF7 DNA methylation 450 K array data indicate unmethylated (black), partial methylation (blue) and methylated (red). **b** GRO-Seq measurements of RNA Polymerase engagement and elongation points from the putative promoters, after E2 stimulation in MCF7 cells. Lower part, ChIP-Seq for binding of ERα and FOXA1 to the putative promoters. **c** qRT-PCR the MIR196A2 response to E2 in MCF7 cells. Qiagen precursor primers were used to detect the precursor miRNA at the specified time points and CT values were normalised to a DMSO vehicle control and the qRT-PCR control of *RNU6b*. **d** MiRNA-Seq RPKM for the precursor miRNAs following E2 addition to MCF7 cells. **e** Luciferase reporter assay measuring the influence of *MIR196A2* putative promoter on the luciferase gene transcription. Measurements are RLU normalised to the renilla plasmid (pRL-TK) acting as a transfection control and to the pGL3/Empty plasmid. Experimental measures are done in triplicate with the experiment repeated, data not shown. E2 oestradiol, H3K4me3 Histone 3 Lysine 4 tri-methylation, H3K27ac Histone 3 Lysine 27 acetylation, H3K27me3 Histone 3 lysine 27 tri-methylation, HMEC Human Mammary Epithelial Cell, GRO-Seq Global run-on sequencing, RPKM reads per kilobase per million, RLU relative light units

We and others have previously demonstrated regulation of *HOXC* genes by oestrogen in breast cancer.^48–52^ Given that *MIR196A* expression strongly correlates with expression of HOXC protein coding genes in breast tumours (Supp Fig. 1), we sought to determine if oestrogen also regulates the HOXC embedded *MIR196A2* precursor gene. Chromatin immunoprecipitation (ChIP-Seq) for RNA polymerase II demonstrates that polymerase binding in the region surrounding the *HOXC10* gene and *MIR196A* gene is dependent on oestrogen in MCF7 cells (Fig. 1a). Global-run-on sequencing (GRO-Seq) is able to measure nascent RNA, assessing changes in transcription with high sensitivity. Analysis of MCF7 GRO-Seq data clearly indicates a dramatic increase in RNA production in the genomic region surrounding *MIR196A2*, peaking at 40 mins following addition of oestradiol (E2) (Fig. 1b). This increase in RNA production from the *HOXC* locus was validated with qRT-PCR from MCF7 cells following addition of E2 (Fig. 1c). The regulation of *HOXC10* by oestrogen has been previously established,^51^ we find similar results which indicate an increase in expression by E2 (Supp Fig. 3). We next analysed data from MCF7 cells where low levels of E2 (1 nM) were used and find a similar pattern of a rapid increase in *pre-MIR196A2* expression (Fig. 1d). Additionally, there was no change in the expression of the HOXB precursor, *pre-MIR196A1*. Taken together this suggests that *MIR196A* is transcriptionally regulated by oestrogen through its *HOXC* precursor, *MIR196A2*.

### Transcriptional regulation of the MIR196A2 precursor gene

To identify the structural elements associated with the transcriptional regulation of *MIR196A2*, histone methylation patterns in the MCF7 breast cancer cell line were assessed. This analysis uncovered putative promoter elements upstream of *MIR196A2* including a shared promoter with *HOXC10* (Fig. 1a). Given the strong association of *MIR196A* and *HOXC10* expression in breast and their co-regulation by oestrogen, it seems likely they may share a common promoter element which we have cloned in three separate elements labelled putative promoter 1 (PP1), Overlap (between PP1 and PP2) and PP2.

Given that *MIR196A2* expression is regulated by oestrogen we hypothesised that its transcription may be controlled by the oestrogen receptor (ER). Using publicly available datasets we established that oestrogen mediated upregulation of *MIR196A2* expression is accompanied by binding of ERα and its pioneer factor FOXA1 to two putative promoter regions, PP1 and PP3, upstream of the *MIR196A2* transcription start site (Fig. 1b).

The putative promoter elements were subsequently cloned into luciferase reporter vectors to assess transcriptional activity. PP1 and PP2 (modestly) increased luciferase gene transcription (Fig. 1e), with the most active promoter in MCF7 cells being PP1 (*HOXC10* promoter).

**Fig. 2:**
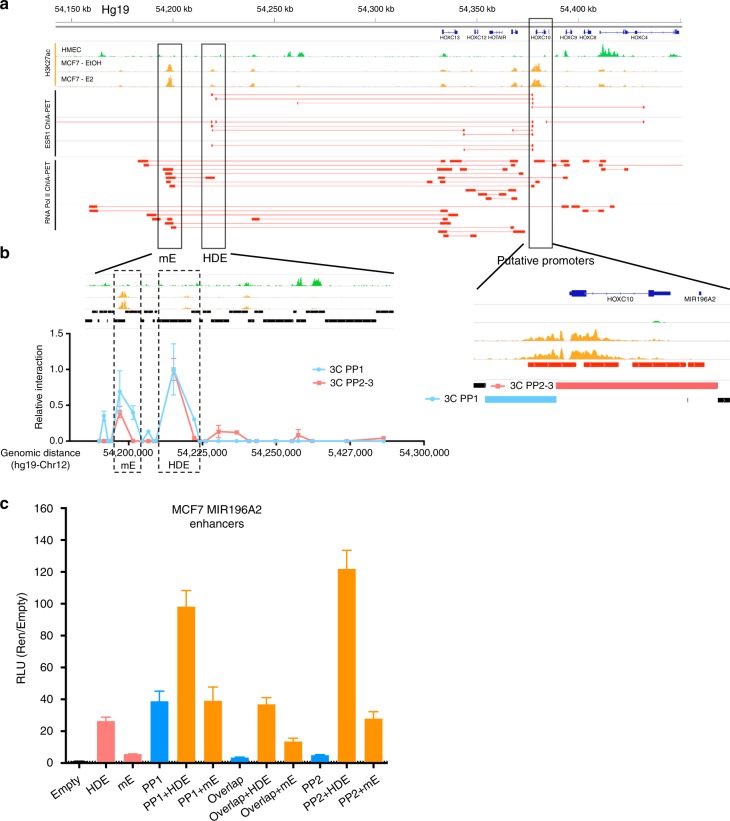
Distal putative enhancer elements of the MIR196A2 putative promoters. **a** Histone modification and ChIA-PET of ESR1 and RNA Pol II in MCF7 cells across the HOXC locus and corresponding gene desert. The histone modification H3K27ac is a measure of regulatory element activity and was assessed in HMEC and MCF7 cells plus or minus E2. ChIA-PET interactions are represented by red lines, solid rectangles indicate the sequenced tag and the two points that were physically interacting and tethered to either ESR1 or RNA Pol II. **b** Zoom-in of mE and HDE (left) and the putative promoter elements (right). Black rectangles indicate the genome fragment sizes post digestion with *Hind*III. Graph is 3C-qPCR for either the 3 C PP1 or 3 C PP2-3 fragments with the *Y*-axis the relative interaction and the *X*-axis the genomic location. All genomic coordinates were based on chromosome 12 in the hg19. **c** Luciferase reporter assay showing the augmentation of HOXC promoters with either mE or HDE, graphed as RLU normalised to the co-transfected control renilla plasmid and to the vector backbone pGL3-Basic. ChIA-PET Chromatin interaction analysis by paired-end tags, HDE *HOTAIR* distal enhancer, mE *MIR196A2* enhancers, PP1/2/3 putative promoter 1

Given that ERα often binds to distal enhancer elements to exert its function, we examined the hypothesis that *MIR196A2* is controlled by long-range transcriptional regulation, mediated by ERα tethered gene looping. Using ChIA-PET (Chromatin Interact Analysis by Paired End Tags) genome-wide chromatin interactions that immunoprecipitate with either ERα or RNA Polymerase II (correlative with active promoters and enhancers), we identified two major sites of interaction with the *MIR196A2/HOXC10* promoters (Fig. 2a). One of these is a previously identified *HOTAIR* enhancer (*HOTAIR* distal enhancer, HDE^49^) and the other a novel interacting partner (*MIR196A2*-Enhancer, mE). Chromosome conformation capture (3C) enzymatic digestion of the HOXC genomic locus results in two fragments covering the *MIR196A2* region. 3C-qPCR analysis demonstrates that both enhancer elements physically interact with each of the *MIR196A2*/*HOXC10* promoter regions (Fig. 2b). Cloning of these fragments downstream of the putative-promoter luciferase reporters clearly demonstrates significant augmentation of transcription for both the PP1 and PP2, with HDE appearing to be the most active in MCF7 cells (Fig. 2c).

Interestingly, a previous study^10^ identified a SNP (rs11614913) and an upstream CpG island that are both associated with a decrease in breast cancer risk. This SNP lies within the *MIR196A2* gene and the CpG island (CpG_Hoffman) is immediately upstream, falling into the 3’ end of the PP3. Analysis of DNA methylation reveals that this CpG island is mostly methylated in non-malignant MCF10A and cancerous MCF7 cells, whilst unmethylated in human mammary epithelial cells (HMEC) (Fig. 1a).

### MIR196A is differentially expressed in breast cancer

**Fig. 3:**
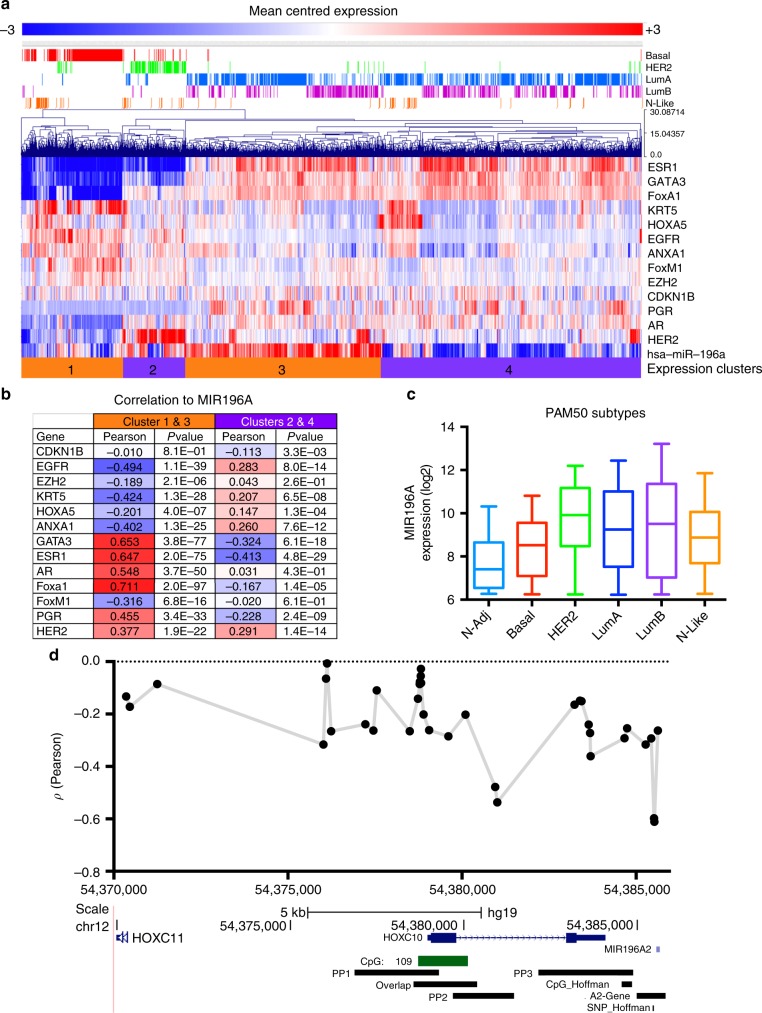
*MIR196A* is differentially expressed in breast cancer. **a** Mean-centred log2-expression of *MIR196A* and commonly utilised breast cancer molecular markers. Expression values were hierarchically clustered and the PAM50 tumour subtypes are indicated above the plot. Expression values are indicated by colour scale bar. **b** Pearson correlation coefficients, and corresponding P-values, for each gene against the expression of *MIR196A* either in the orange or purple clusters. **c** Intensity values for the expression of *MIR196A* across the five molecular subtypes, PAM50. **d** Correlation of *HOXC* methylation to expression of *MIR196A* for CpG dinucleotides upstream of its gene. Data was sourced from the METABRIC cohort^40,41^ for **a**, **b** and **c**. Methylation and expression data for **d** was sourced from the TCGA cohort of breast tumours.^36^ Basal (*n* = 179), HER2 (HER2-enriched, *n* = 112), LumA (Luminal A, *n* = 568), LumB (Luminal B, *n* = 354), N-Adj (Normal-adjacent, *n* = 116), N-Like (Normal-like, *n* = 82)

Since *MIR196A* is regulated by ERα, we investigated its expression patterns in relation to commonly utilised molecular markers of breast tumours (Fig. 3a). This analysis identified four distinct clusters of *MIR196A* expression (Clusters 1–4). Interestingly clusters 1 and 3 show a strong correlation to expression of hormone receptors (HR) (*AR, ERα, PGR, HER2*) and HR cofactors (Fig. 3b). In contrast, clusters 2 and 4 have significant negative correlation to expression of *ERα*, *PGR*, *FOXA1* and *GATA3*, whilst associating with *EGFR* and *HER2*. This expression is further defined by the PAM50 intrinsic subtypes where *MIR196A* is strongly expressed in the HER2 subtype, whist in the luminal A and B subtypes expression is very dynamic (Fig. 3c).

DNA methylation accumulates within promoters at CpG islands to suppress gene expression through inhibition of transcription factor binding.^53^ To predict further regulators of MIR196A expression in clusters 2 and 4 where negative correlation to ESR1 is seen, we investigated the DNA methylation of our putative promoter elements. The majority of upstream CpGs show a negative correlation to *MIR196A* expression, which the strongest correlation seen to sites within PP2 and A2-Gene (Fig. 3d). Utilising ENCODE TF ChIP-Seq data and motif sites from JASPAR, we identified 49 TFs binding within 100 bp of these methylation sites (Supp Figs. 4 and 5, Supp Table 3). Of the 49 TFs, 18 factors exhibit significant positive correlation in clusters 2 and 4, while 9 are significantly negatively corelated to *MIR196* *A* expression (Supp Fig. 6A, Supp Table 3). Interestingly, a cluster of factors (CEBPA, CEBPB, EBF1, EGR1, EGR2, EZH2, JUN, KLF4, KLF5, PPARG, RXRA) presents as highly interconnected through protein-protein interactions and transcriptional regulation and appears largely independent of ERα (Supp Fig. 6B, blue oval). These data suggest that in breast cancer, an interconnected group of transcription factors may influence expression of *MIR196A* independent of ERα.

### MIR196A is a biomarker of breast cancer progression

**Fig. 4:**
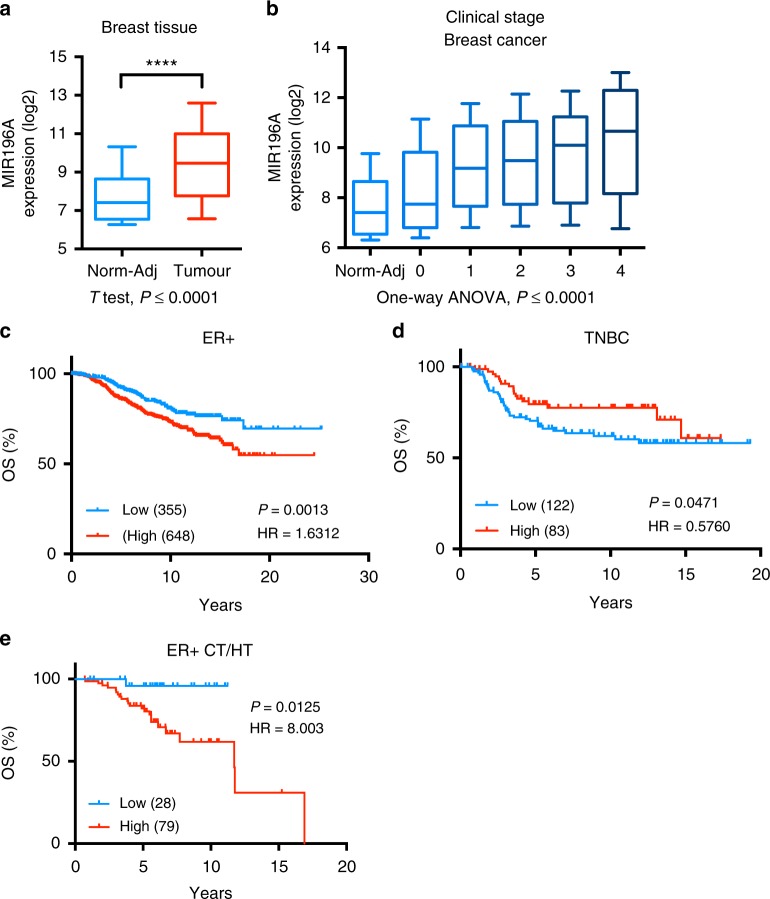
*MIR196A* is a biomarker of breast cancer progression. **a** Log2 miR-Array intensity for the expression of *MIR196A* in normal adjacent tissue and breast tumours. Two-tailed *T*-test with *p* value < 0.0001 (****). **b** Log2 intensity for the expression of *MIR196A* in normal adjacent tissue and tumours at stages 0 to 4. A One-Way ANOVA was used to find a significant trend with a *p*-value = < 0.0001. **c, d** Kaplan-Meier curves stratifying OS of breast tumours by expression of *MIR196A* for patients with ER+ or TNBC. **e** Kaplan-Meier survival curves for patients with ER+ disease, treated with both CT and HT. Expression and survival data sourced from METABRIC.^40,41^ Log-rank *p*-value (P) and hazard ratios (HR) displayed. CT Chemotherapy, HT hormone therapy, Norm-Adj Normal Adjacent, OS overall survival and TNBC triple-negative breast cancer

To further explore the expression of *MIR196A* in breast cancer, we utilised expression data from the METABRIC cohort of breast tumours.^40,41^ Expression analysis of this miRNA indicate that it is significantly over-expressed in breast tumours compared to normal adjacent tissue and over-expression is associated with an increase in tumour stage (Fig. 4a, b). Interestingly, high expression of *MIR196A* is associated with a poor survival in oestrogen receptor positive (ER+) breast cancer, whilst high expression associates with a better outcome in triple-negative breast cancer (TNBC) over the first 5 to 10 years following initial diagnosis (Fig. 4c, d).

Using *MIR196A* expression, overall survival of ER + tumours responding to both hormone therapy (HT) and chemotherapy (CT) was stratified (Fig. 4e). Women with low *MIR196A* expression had exhibited a high rate of survival (>95% at 10 years, HR = 8.003, *P*-value = 0.0125), whilst most women within the high expression group died within 17 years (61% at 10 years).

Given that *MIR196A* is regulated in part by oestrogen, and the disparity in prognostication of ER+ and TNBC, we investigated the effects of menopause on the stratification of survival for ER+ women. The effects of menopause on the human breast are largely unknown, however serum levels of oestrogen and progesterone dramatically reduce post menopause. In pre-menopausal women, high expression of *MIR196A* is associated with improved overall survival in ER+ disease (HR = 0.463, *P*-value = 0.0288) (Table 1, Supp Fig. 7A). Multivariate analysis demonstrates that *MIR196A* is one of the few significant biomarkers for ER+ tumours arising before menopause. In post-menopausal women, all tested biomarkers were significant in ER+ disease, including *MIR196A*, however high expression is now associated with decreased overall survival (HR = 1.847, *P*-value = 0.0005) (Table 1, Supp Fig. 7B). A similar trend was also observed in TNBC, where in pre-menopausal women, *MIR196A* high expression correlates with a better outcome (Supp Fig. 7C), stratification in post-menopausal women however, found no significant trend (Supp Fig. 7D).

**Table 1:** Menopause effects the stratification of patient survival by *MIR196A* expression in ER+ disease

ER+ pre-menopausal						
Condition	Univariate Cox-proportional hazard ratio			Multivariate Cox-proportional hazard ratio (stepwise)		
	HR	(95% CI)	*P*-value	HR	(95% CI)	P-Value
HER2 (high vs. low)	2.695	1.2479–5.8213	0.0120	3.352	1.3483–8.3325	0.0096
MIR196A (high vs. low)	0.463	0.2325–0.9202	0.0288	0.342	0.1534–0.7623	0.0091
Tumour grade (1,2,3)	1.638	0.9988–2.6844	0.0517			
Tumour stage (0–4)	1.652	0.9937–2.7472	0.0541			
Lymph node (+, −)	1.857	0.9836–3.5069	0.0575			
Size (T1, T2, T3)	1.470	0.9518–2.2688	0.0840	1.798	1.0893–2.9693	0.0225
PGR (high vs. low)	0.552	0.2770–1.0978	0.0919			
Age at diagnosis	0.985	0.9261–1.0469	0.6221			

ER+ post-menopausal						
Lymph node (+, −)	2.739	2.0075–3.7358	<0.0001	1.720	1.1510–2.5711	0.0085
Tumour stage (0–4)	2.363	1.8658–2.9926	<0.0001	1.519	1.0557–2.1868	0.0251
Size (T1, T2, T3)	1.866	1.4837–2.3461	<0.0001	1.560	1.1146–2.1832	0.0099
Tumour grade (1,2,3)	1.822	1.4057–2.3622	<0.0001	1.455	1.0926–1.9385	0.0107
MIR196A (high vs. low)	1.847	1.3065–2.6110	0.0005	1.599	1.0806–2.3652	0.0195
HER2 (high vs. low)	2.165	1.3982–3.3521	0.0006	2.210	1.3624–3.5847	0.0014
PGR (high vs. low)	0.636	0.4708–0.8594	0.0034			
Age at diagnosis	1.023	1.0059–1.0403	0.0086			

The overall survival of patients with ER+ disease was stratified by *MIR196A*, HER2, or PGR expression or commonly utilised clinical markers. On the left is the univariatie cox-proportional hazard ratios for each condition, and the right the multivariate cox-proportional hazard model and the conditions which contribute to the most significant model. Expression and survival data sourced from METABRIC^40,41^

*CI* confidence interval, *HR* hazard ratio

### Therapeutic resistance leads to increases in MIR196A expression

**Fig. 5:**
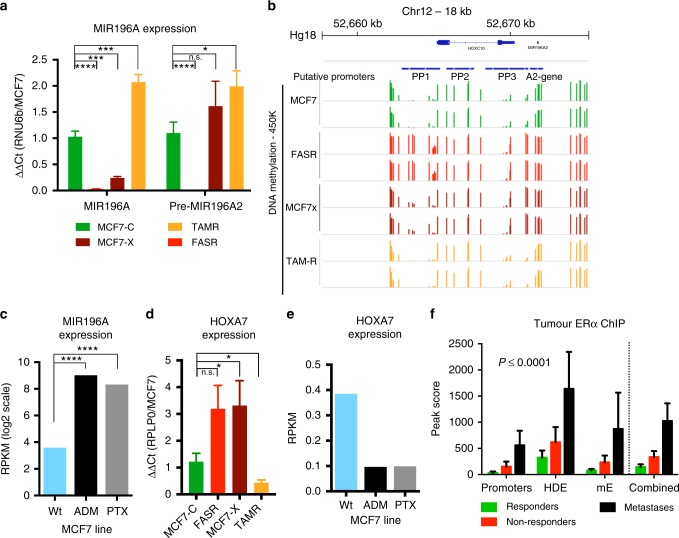
Therapeutic resistance leads to an increase in *MIR196A* expression. **a, d** qRT-PCR of the relative expression for the mature *MIR196A* and precursor *MIR196A2* transcripts and *HOXA7* in MCF7 derived cell line models of endocrine therapy resistance. miRNA expression values are normalised to the expression of *RNU6b* and the MCF7-C cell line, *HOXA7* expression was normalised to *RPLP0* and MCF7-C expression. Error bars are the standard deviation of two technical replicates and four biological replicates. **b** Corresponding DNA methylation for MCF7 derived cell lines, as measured by 450 K methylation array, for the *MIR196A2* genomic region. **c, e** Log2 RPKM expression of *MIR196A* and *HOXA7* in MCF7 wild-type and ADM and PTX derived resistance cell lines. **f** Peak scores for the binding of ERα to *MIR196A2* regulatory elements in ER+ breast tumours. Peak scores were generated using MACS, normalised to the Input control for the ChIP-Seq library. Peak scores are the average for 9 responders, 9 non-responders and 3 metastases. Data is sourced from Ross-Innes et al.^30^ ADM Adriamycin/doxorubicin, FASR Fulvestrant resistant, MACS model-based analysis for ChIP-Seq, MCF7-C Control, MCF7-X Oestrogen deprived, PTX paclitaxel, RPKM reads-per-kilobase-per-million, TAMR Tamoxifen resistant

TNBC is resistant to hormone-based therapies and HR+ disease often becomes resistant to anti-oestrogen treatment. Using established models of HR+ disease resistance we found that *MIR196A* expression is significantly increased in tamoxifen resistant MCF7 cells (TAMR) whilst it is almost depleted in acquired fulvestrant resistance (FASR) (Fig. 5a). These expression patterns match changes in DNA methylation to the *HOXC10*/*MIR196A2* promoters in these same cells (Fig. 5b). For HR+ resistant tumours the only remaining therapeutic options are radiotherapy and chemotherapy. Using RNA-Seq data for cell line models of resistance to adromycin (ADM) and paclitaxel (PTX), two commonly used chemotherapeutics, *MIR196A* expression again increases in resistant cell lines compared to the treatment sensitive cell line (Fig. 5c). These data suggest an intrinsic requirement for elevated *MIR196A* expression in HR+ tumour resistance.

Several HOX genes are validated targets of *MIR196A* (*HOXA7*, *HOXB8*, *HOXC8* and *HOXCD8*) HOXA7 shows the greatest negative correlation to *MIR196A* in human breast cells (Supp Fig. 2A). Expression of *HOXA7* strongly mirrors that of *MIR196A* in the panel of endocrine resistant MCF7 sublines (Fig. 5d). Additionally, expression of *HOXA7* decreases in the ADM and PTX resistant lines (Fig. 5e), in contrast to the increase in *MIR196A* expression. In these models of therapeutic resistance, *MIR196A* may be reducing expression of *HOXA7*.

Utilising ERα ChIP-Seq performed in human patients with HR+ disease,^30^ binding sites for ERα were identified in the genomic region of *MIR196A*. This tumour cohort contains three groups of tumours, (1) tumours from women who respond to HR therapy, (2) those who do not and (3) metastases from resistant tumours. An increase in ERα occupancy is seen at both enhancer and promoter regions of *MIR196A* in non-responders and metastases (Fig. 5f). The increased genome-wide ERα binding in the more resistant tumours was shown by the authors to associate with changes to expression patterns crucial for the resistant tumour to survive therapy and become resistant.

## Discussion

The expression of *MIR196A* in breast cancer is both dynamic and complex. In this paper, we have elucidated important elements, factors and mechanisms controlling the transcriptional regulation of *MIR196A* and shown that changes in regulation are associated with breast cancer progression and therapeutic resistance.

Several studies have demonstrated regulation of *HOXC* genes by oestrogen.^49,51,54^ The majority of *HOXC* genes are lowly expressed in breast luminal epithelial cells (BLEC), where ERα is most highly expressed and cells are responsive to oestrogen. The regulation of *HOXC* genes by ERα may be specific to cancer cells through an acquired mechanism of regulation. Several studies have shown that enhancers that are normally repressed can become activated in cancer^55,56^ and given the extensive chromatin looping between the *HOXC* locus and its adjacent gene desert, this seems the likely mechanism for cancer expression.

We have previously demonstrated that long-range regulation of *HOXC* genes occurs in breast cancer and is influenced by ERα and its associated cofactors.^49^ *HOX* gene expression is tightly controlled in a spatiotemporal manner to ensure proper axial formation along the anterior-posterior axis during embryonic development.^57^ Within the cell types of the human breast, *HOX* gene expression appears dynamic and the association between *MIR196A* and *HOXC* genes is not significant. The strong correlation in expression of all *HOXC* genes in breast tumours with *MIR196A* is in stark contrast to expression in normal tissues. Several instances have been described regarding the influence of multiple distal enhancers on gene expression, such as the well characterised locus-control-region (LCR) of the Beta-globin genes or the c-Myc enhancers active across multiple cancer types.^45,58–60^ Given the extensive interactions between this locus and its adjacent gene desert, we hypothesise that a consorted effort of multiple enhancers is responsible for the overexpression of these genes in cancer possibly driven by extensive binding and activity of ERα. To explore this hypothesis a high resolution chromatin interaction analysis of this region in breast cancer cells would be required, such as 5C^61^ or NG Capture-C,^62^ coupled with ERα ChIP-Seq and ChIA-PET.^29^ In addition, we see an increase in *MIR196A2* expression in response to low-dose E2, suggesting a direct influence by ERα that is further increased at higher dosages of 10 nM in our qRT-PCR assays. It would be interesting to explore how low dosages of E2 influence distal enhaner elements and if more oestrogen is required for enhancer-promoter activity.

Whilst this manuscript was in preparation new data has come to light which corroborates our conclusions. Jiang et al.^63^ demonstrate that the mature *MIR196A* transcript positively responds to oestrogen stimulation in MCF7 cells, and this is mediated by upstream ERα binding. This binding peak falls within PP3. Whilst we show that PP3 is not able to increase luciferase expression in a luciferase reporter assay, the binding of ERα may be important for the activity of the *HOXC10* and *MIR196A2* promoters. In our data we see a time delay in the processing of the precursor *MIR196A2* gene into mature *MIR196A*, suggesting a second mechanism of regulation post-transcriptionally. Evidence suggests that the levels of mature miRNAs are more reliant the microprocessor complex and that individual miRNAs vary significantly in there maturation and stability.^64^

Previous genetic association studies have demonstrated that the SNP (rs116149130) within the precursor gene, *MIR196A2*, confers a reduced risk of breast cancer incidence.^10,65,66^ This SNP is found within the *MIR196A-3p* sequence of the *MIR196A2* precursor gene. Hoffman and colleagues^10^ demonstrated that rs116149130 reduces microRNA maturation thereby reducing expression of the mature miRNA. They also identified that an upstream CpG island is associated with reduced risk when hypermethylated. Here we show that this upstream CpG island lies within the transcriptionally active region of *HOXC10* and *MIR196A2* as observed through GRO-Seq. Interestingly, this CpG island is completely methylated in models of oestrogen deprivation and fulvestrant treatment, but not in tamoxifen resistant cells. DNA methylation is most commonly associated with repressed transcription,^67^ hypermethylation of this region in a transcriptional high region may severely impair expression. Given that various transcription factors strongly influence transcription in endocrine resistant breast cancer, these data suggest that binding of ERα accompanied by cofactors may be needed to maintain low methylation levels and active transcription in breast cancer.^68–72^

Using hierarchical clustering of breast tumour RNA-Seq data, we observed two distinct expression patterns associated with *MIR196A* expression. Interestingly, DNA methylation at several sites within the *HOXC* locus negatively correlates with the expression of this miRNA, supporting the notion of DNA methylation as a repressive epigenetic modification in this context.^67^ We demonstrated that several transcription factors that bind to these differentially methylated regions strongly associate with *MIR196A* expression in breast cancer, even in those tumours with show a negative correlation to ERα expression. These transcription factors appear to influence the expression of one another and in some cases form protein-protein complexes. Further investigations should aim to fully elucidate the role of this network and its influence on *MIR196A* expression.

High expression of *MIR196A* is a biomarker of poor prognosis in ER+ tumours, especially in those patients resistant to therapy. Expression of *MIR196A* increases in response to tamoxifen and chemotherapeutic agents in oestrogen responsive MCF7 cells. This increase in expression is associated with loss of DNA methylation within the promoter regions of the miRNA. In poor responders with ER+ tumours, *HOXC* enhancer elements appear to more readily bind the ER. These data raise the possibility that the pathway to resistance to therapy in ER+ tumours involves the de-repression and over-activation of promoter and enhancer elements. This is commonly seen throughout cancer,^56,73,74^ with suggestions that enhancer disruption can revert cells to a non-terminally-differentiated state a common hallmark of tumourigenesis. *HOX* genes are essential in embryonic development, these genes would be a valuable asset for any tumour cell to use to sustain a stem-cell like state.^75,76^

Breast cancer incidence and relative subtype changes after menopause.^77,78^ In women younger than 45, luminal breast tumours account for 33–44%.^79,80^ This increases to 70–72% in women older than 65. In contrast, basal-like tumours are more common in younger women, suggesting a switch or evolution in the factors driving cancer following menopause, most likely related to the decline in oestrogen production. It is then interesting to note that higher expression of *MIR196A* associates with good outcome in pre-menopausal women with ER+ tumours, and a poor outcome of ER+ tumours following menopause. Given the strong involvement of *HOX* genes in development, we hypothesise that there is a change in the regulation and expression of these genes through and following menopause, which in turn impacts their contribution to the development of certain breast cancer subtypes.

*MIR196A* is a dynamically expressed miRNA in both normal mammary cells and breast tumours. This miRNA is a possible biomarker for the progression of breast tumour to becoming resistant to therapy. Future studies should aim to uncover the purpose of increase *MIR196A* expression and if it is required for development of resistance alone or in combination with other *HOXC* genes.

## Supplementary information

Supp Figure 2. MIR196A is highly expressed in breast stem cells

Supp Figure 3. Oestrogen induces expression of TFF1 and HOXC10 in MCF7 cells

Supp Figure 4. Transcription factor binding within Putative Promoter Element 2

Supp Figure 1. MIR196A expression correlates with HOXC genes in breast cancer

Supp Figure 6. A network of transcription factors binds to MIR196A putative promoters

Supp Figure 5. Transcription factor binding within the A2-Geneputative promoter

Supp Figure 7. Menopausal status effects the stratification of overall survival in ER+ and TNBC patients by MIR196A expression

Supp Table 1

Supp Table 2

Supp Table 3

## Data Availability

Requests for data and reagents can be made by contacting the corresponding or senior authors.
